# Acupuncture treatment of glaucoma based on radar plots: A protocol for an overview of systematic reviews

**DOI:** 10.1097/MD.0000000000027261

**Published:** 2021-09-24

**Authors:** Yu Liu, Zhangxin Li, Ruixin Gao, Wei Wang, Tingting Cao, Suhong Ma

**Affiliations:** a Department of Ophthalmology, Cangzhou Central Hospital, Cangzhou, Hebei Province, China; b Department of Ophthalmology, Qinghai Red Cross Hospital, Xining, Qinghai Province, China; c Department of Ophthalmology, Kunming Municipal Hospital of Traditioal Chinese Medicine, Kunming, Yunnan Province, China; d College of Acupuncture, Massage and Rehabilitation, Yunnan University of Chinese Medicine, Kunming, Yunnan Province, China.

**Keywords:** acupuncture, a measurement tool to assess systematic reviews 2, glaucoma, overview, preferred reporting items for systematic review and meta-analysis, radar plots

## Abstract

**Background:**

Glaucoma is the second most blinding eye disease in the world. Currently, lowering the intraocular pressure through various methods is the main treatment of glaucoma. Acupuncture has been effectively and safely used in the treatment of glaucoma. However, the evidence for the efficacy of acupuncture in the treatment of glaucoma is controversial, leading to inconsistent findings from systematic evaluations at abroad and home. Therefore, this protocol aims to provide a multivariate evaluation on the quality of evidences from current systematic reviews (SRs) and/or meta-analyzes (MAs) of acupuncture in the treatment of glaucoma, and literature quality, thus providing an intuitive and reliable evidence synthesis and basis for clinical decision making.

**Methods:**

MAs/SRs about the acupuncture treatment of glaucoma will be searched online, including Chinese Biomedical Literature Database (CBM), China Science and Technology Journal Database (VIP), China National Knowledge Infrastructure (CNKI), WanFang Database (WF), Web of Science, Embase, PubMed, and Cochrane Library. Two investigators will independently screen literatures according to inclusion and exclusion criteria and extract data. A multivariate evaluation of the included literature will be performed by depicting radar plots in 6 aspects as follows: Year of publication, study type, SRs assessment through the Assessment of Multiple Systematic Reviews 2 (AMSTAR 2), literature quality assessment through the Preferred Reporting Items for Systematic Reviews and Meta-analyses (PRISMA), homogeneity, and publication bias. The Grading of Recommendations Assessment, Development, and Evaluation evidence quality assessment tool will be used to grade and evaluate the quality of outcome indicators of the included literatures.

**Results:**

This study will be submitted for publication in a peer-reviewed journal.

**Conclusion:**

We would like to provide a visual and scientific approach for clinical decision making of acupuncture treatment of glaucoma through a accessible and useful assessment of systematic reviews.

## 1 Introduction

Glaucoma is a serious and blinding eye disease characterized by visual field loss and optic nerve atrophy.^[1–3]^ The World Health Organization (WHO) proposed that glaucoma is the second leading cause of blindness.^[1,4]^ Epidemiological data based on the estimated national population in 2020 have shown that the number of glaucoma patients in the world has reached 79.6 million in 2020, involving 6 million in China.^[5]^ The damage to visual function caused by glaucoma is irreversible, leading to extremely serious consequences.^[6,7]^ Therefore, early detection, diagnosis, treatment and prevention of glaucoma are of great significance to improve its prognosis.

Western medical treatment of glaucoma includes drugs, laser treatment and surgery, all of which can be effective, but a long-term use of Western medicine can cause adverse events with varying degrees.^[8,9]^ Traditional Chinese Medicine (TCM) treatment has significant advantages in controlling optic nerve damages in glaucoma patients with fewer adverse events, which is effective in protecting vision loss after stabilizing intraocular pressure.^[10–12]^ Therefore, it is important to study the mechanism of the protective effect of TCM treatment on the optic nerve of glaucoma.

In TCM theory, glaucoma is a disease of Qingmang and the five wind-type internal obstruction.^[13,14]^ Ancient medical records have proven the effectives of acupuncture in the treatment of glaucoma. There are no significances in lowering intraocular pressure and alleviating visual field defects between eye drops and acupuncture.^[15]^ However, acupuncture presents a higher efficacy, simpler procedures and less adverse events in the treatment of glaucoma.^[16,17]^ Acupuncture treatment provides an economical and convenient option for glaucoma patients, especially those who are intolerant to drug therapy.^[18]^

Through literature review, several systematic reviews (SRs) and meta-analyzes (MAs) at abroad and home have assessed acupuncture treatment of glaucoma, although the results and literature quality are inconsistent.^[15,19–22]^ Systematic evaluations are recognized as the best evidence synthesis studies in clinical decision making. High-quality systematic evaluations provide a basis for decision-making by clinicians, patients, and other stakeholders. In contrast, low-quality systematic evaluations are likely to mislead decision makers. This study will screen out relevant SRs/MAs on acupuncture treatment of glaucoma in recent years, and collect data for analyses. In detail, the literature quality of SRs/MAs on needle acupuncture treatment of glaucoma will subjected to a multivariate evaluation in the following terms: Year of publication, study type, SRs assessment through a measurement tool to assess systematic reviews 2 (AMSTAR 2), literature quality assessment through preferred reporting items for systematic review and meta-analysis, homogeneity and risk of publication bias. In addition, the quality of evidence for their outcome indicators will be re-graded using grading of recommendations assessment, development, and evaluation that provides an intuitive and reliable evidence synthesis and decision basis for clinical acupuncture for glaucoma, and facilitates future adjustments in the diagnosis and treatment protocols of acupuncture for glaucoma.

## 2 Methods

### 2.1 Study registration

The protocol will be written in accordance with the Preferred Reporting Items for Overview of SRs (PRIO-harms).^[23]^ This protocol has been registered with Open Science Framework Registries (registration number: DOI 10.17605/OSF.IO/FYCZB).

### 2.2 Inclusion criteria for study selection

#### 2.2.1 Type of studies

Published SRs reported in Chinese or English language, and the included studies are randomized controlled clinical trials (RCTs) for acupuncture treatment of glaucoma.

#### 2.2.2 Types of participants

Glaucoma patients. The course of disease, age, sex, and race for glaucoma patients will be unlimited.

#### 2.2.3 Types of interventions

##### 2.2.3.1 Experimental interventions

Acupuncture treatment, including acupuncture, electroacupuncture, fire needle, auricular acupuncture, catgut embedding, auricular therapy, acupressure, acupoint injection, or any combination of the above.

##### 2.2.3.2 Control interventions

Placebo, sham acupuncture, drugs, or other conventional treatments like sham treatment.

#### 2.2.4 Types of outcome measures

Total effective rate, visual field change and intraocular pressure change level.

### 2.3 Exclusion criteria

1. Duplicate publications;
2. Protocols, meetings, abstracts, non-full text, expert experience, animal experiment;
3. Non-RCTs included in MAs/SRs

### 2.4 Data sources

MAs/SRs about the acupuncture treatment of glaucoma published before August 2021 will be searched online, including CBM, VIP, CNKI, WF, Web of Science, Embase, PubMed, and Cochrane Library. A combination of subject terms and key words will be used for screening out eligible literature as much as possible. References of eligible literatures will be manually searched to avoid missing data.

### 2.5 Searching strategy

Searching strategies using the Pubmed were illustrated in Table 1, and literature search in other online databases will be similarly conducted.

**Table 1:** Search strategy in PubMed database.

Number	Search terms
#1	Glaucoma[MeSH]
#2	Glaucomas[Title/Abstract]
#3	OR/1–2
#4	Acupuncture[MeSH]
#5	Acupuncture Therapy[Title/Abstract]
#6	Acupuncture Points[MeSH]
#7	Acupoints[Title/Abstract]
#8	Acupoint[Title/Abstract]
#9	Acupuncture Point[Title/Abstract]
#10	Point, Acupuncture[Title/Abstract]
#11	Points, Acupuncture[Title/Abstract]
#12	Acupuncture Therapy[MeSH]
#13	Therapy, Acupuncture[Title/Abstract]
#14	Acupuncture;Medicine, Chinese Traditional[MeSH]
#15	Electroacupuncture[MeSH]
#16	Acupuncture, Ear[MeSH]
#17	Acupuncture, Auricular[Title/Abstract]
#18	Auricular Acupuncture[Title/Abstract]
#19	Ear Acupuncture[Title/Abstract]
#20	Acupunctures, Auricular[Title/Abstract]
#21	Acupunctures, Ear[Title/Abstract]
#22	Auricular Acupunctures[Title/Abstract]
#23	Ear Acupunctures[Title/Abstract]
#24	Fire needle[Title/Abstract]
#25	Warm acupuncture[Title/Abstract]
#26	Blood-pricking[Title/Abstract]
#27	Acupuncture-moxibustion[Title/Abstract]
#28	Acupoint[Title/Abstract]
#29	Moxa needle[Title/Abstract]
#30	Auricular needle[Title/Abstract]
#31	Ear acupuncture[Title/Abstract]
#32	Moxibustion[Title/Abstract]
#33	Abdom^∗^ acupuncture[Title/Abstract]
#34	Embedded thread therapy[Title/Abstract]
#35	Embedding thread[Title/Abstract]
#36	Catgut implantation at acupoint[Title/Abstract]
#37	Acupuncture Points[MeSH]
#38	Point[Title/Abstract]
#39	OR/4-38
#40	Systematic Review [Publication Type]
#41	Systematic Reviews as topic[MeSH]
#42	Network Meta-Analysis[MeSH]
#43	Meta-analysis [Publication Type]
#44	Meta-analysis as topic[MeSH]
#45	Systematic review[Title/Abstract]
#46	Meta-analysis[Title/Abstract]
#47	OR/40-46
#48	#3 AND #39 AND #47

### 2.6 Literature screening and data extraction

In literature screening, 2 investigators will independently read the titles and abstracts of the retrieved studies at first, and determine the inclusion or exclusion, and then the full text will be further reviewed for in-depth screening. In case of disagreement, a third reviewer will participate in a consensus meeting. Excel 2016 will be used to record data collected from eligible literatures, including:

1. Basic information, including first author, year of publication, country of the study, number of included literatures, sample size, tools for evaluating risk of bias, intervention/control measures, and meta-analysis outcome indicators;
2. Radar plots for multivariate data visualization, including year of publication, study type, AMSTAR 2 results, Preferred Reporting Items for Systematic Review and Meta-analysis (PRISMA) scores, homogeneity, and publication bias. Data extraction and summarization will independently performed by 2 investigators mentioned above.

**Figure 1:**
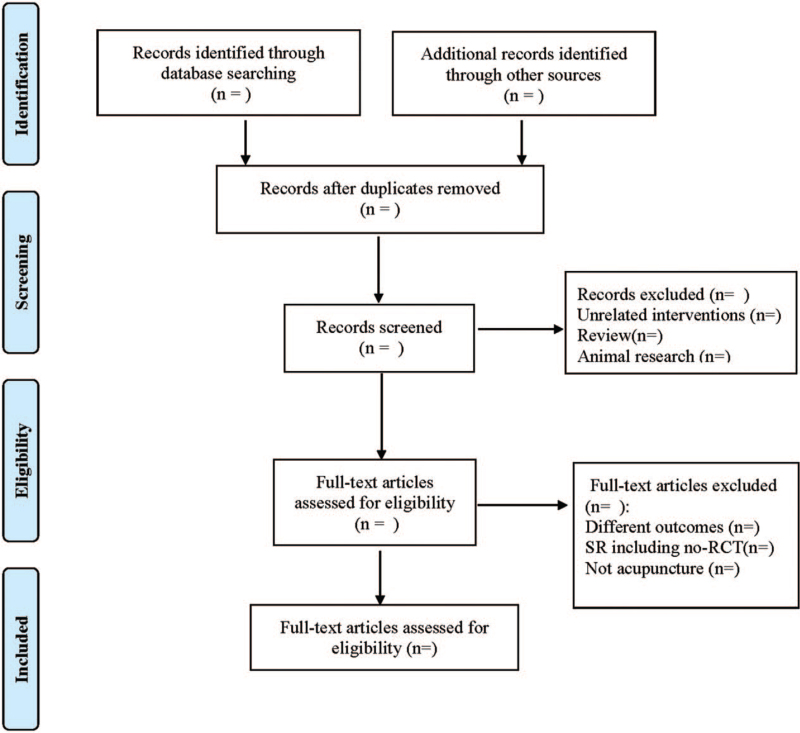
Flow diagram of study selection process.

Any disagreement will be solved through discussion and agreement. The PRISMA flow chart of studies for SRs/ MAs was shown in Figure 1.

### 2.7 Data synthesis

We will provide a narrative description of the included SRs/MAs and create tables to detail the upper key components and outcomes. In addition, we will integrate these data to provide a composite treatment efficacy of all SRs, including scores for each outcome indicator. Subgroup analyses will be performed to compare acupuncture monotherapy or a combined treatment involving acupuncture. If necessary, a meta-analysis of the results will be performed using RevMan 5.3 software.

### 2.8 Subgroup analyzes

Subgroup analyses will be conducted based on different types of acupuncture methods and duration of intervention.

### 2.9 Principles of depicting radar plots

The scores of each evaluation item will be rank-transformed according to the statistical processing of ranked data. The total number of literatures will be taken as the highest rank value, while the remaining evaluation items will be ranked from high to low based on the descending order of the total number of literatures, and the mean value of the six dimensional aspects will be calculated as the mean rank score.

### 2.10 Evaluation of the quality of the included systematic reviews/meta-analyzes

#### 2.10.1 Assessment of methodological quality of included systematic reviews/meta-analyzes

The methodological quality of SRs/MAs reporting the acupuncture treatment of glaucoma will be evaluated according to the 11 items of the AMSTAR 2 scale ranging from 0 to 11 scores.^[24]^ Each entry in the AMSTAR 2 scale will be scored as 1 for normative and correct use and 0 for non-use or misuse.

#### 2.10.2 Report quality of included reviews

The PRISMA tool will be used to evaluate the quality of literatures included in the SRs/MAs.^[25]^ It is a 27-point scale with each item graded 1 point for standardized and correct use, 0.5 point for incomplete use, and 0 point for non-use or misuse.

#### 2.10.3 Quality of evidences in included systematic reviews/meta-analyzes

The quality of evidences for each outcome indicator included in SRs/MAs will be evaluated by the grading of recommendations assessment, development, and evaluation tool.^[26]^ Limitations, inconsistency, non-directivity, imprecision, and publication bias will be used as downgrading factors. Evidence quality will be rated as high quality with no downgrading, medium quality with 1 downgrade, low quality with 2 downgrades, and very low quality with 3 or more downgrades.

### 2.11 Homogeneity

*I*^*2*^ and chi-square tests will be used to assess the heterogeneity of included literatures. More than half of the outcome indicators in the included literatures with *P* ≥ .01 and *I*^2^ ≤ 50% will be considered as high homogeneity.

### 2.12 Published bias

A low risk of publication bias will be considered if it was assessed using funnel plots or other methods in the recruited literatures^[27,28]^; Otherwise, it will be considered as a high risk of publication.

### 2.13 Ethics and dissemination

Since the program does not include the recruitment of patients and the collection of personal information, it does not require the approval of the Ethics Committee.

## 3 Discussion

High-quality SRs/MAs are one of the most important sources of evidence-based medicine for obtaining the best evidences, which also provide references for clinical decision making in acupuncture. Although a large number of SRs/MAs on acupuncture treatment of glaucoma have been published in peer-reviewed journals, their reporting and methodological qualities remain unclear and conflicting. This protocol aims to re-evaluates the extant SRs/MAs, thus providing an evidence-based basis for the treatment of glaucoma.

## Author contributions

**Conceptualization:** Suhong Ma, Yu Liu.

**Data curation:** Zhangxin Li.

**Formal analysis:** Zhangxin Li.

**Funding acquisition:** Suhong Ma.

**Investigation:** Zhangxin Li.

**Methodology:** Zhangxin Li, Ruixin Gao.

**Project administration:** Suhong Ma.

**Resources:** Ruixin Gao.

**Software:** Ruixin Gao, Wei Wang.

**Supervision:** Suhong Ma.

**Validation:** Wei Wang, Tingting Cao.

**Visualization:** Wei Wang, Tingting Cao.

**Writing – original draft:** Suhong Ma, Yu Liu.

**Writing – review & editing:** Suhong Ma, Yu Liu.
